# Acceptance of COVID-19 Vaccination in the Elderly: A Cross-Sectional Study in Southern Italy

**DOI:** 10.3390/vaccines9111222

**Published:** 2021-10-21

**Authors:** Francesca Gallè, Elita Anna Sabella, Paolo Roma, Giovanna Da Molin, Giusy Diella, Maria Teresa Montagna, Stefano Ferracuti, Giorgio Liguori, Giovanni Battista Orsi, Christian Napoli

**Affiliations:** 1 Department of Movement Sciences and Wellbeing, University of Naples “Parthenope”, 80133 Naples, Italy; giorgio.liguori@uniparthenope.it; 2 Inter-University Research Centre “Population, Environment and Health”, University of Bari Aldo Moro, 70121 Bari, Italy; elita.sabella@uniba.it (E.A.S.); giovanna.damolin@uniba.it (G.D.M.); 3 Department of Human Neurosciences, “Sapienza” University of Rome, 00185 Rome, Italy; paolo.roma@uniroma1.it (P.R.); stefano.ferracuti@uniroma1.it (S.F.); 4 Department of Biomedical Science and Human Oncology, University of Bari Aldo Moro, 70124 Bari, Italy; giusy.diella@uniba.it (G.D.); mariateresa.montagna@uniba.it (M.T.M.); 5 Department of Public Health and Infectious Diseases, “Sapienza” University of Rome, 00185 Rome, Italy; giovanni.orsi@uniroma1.it; 6 Department of Medical Surgical Sciences and Translational Medicine, “Sapienza” University of Rome, 00189 Rome, Italy; christian.napoli@uniroma1.it

**Keywords:** COVID-19, immunization campaign, elderly, vaccine acceptance, *green pass*

## Abstract

In Italy, at the end of 2020, a voluntary immunization plan against COVID-19 was introduced, involving elderly among the first target categories. The aim of this study was to assess, through an online questionnaire, the acceptance of COVID-19 vaccination in a sample of older adults from southern Italy. Of a total of 1041 respondents (41.7% males, mean age 76.6 ± 6.5), 965 (92.7%) were vaccinated or willing to be vaccinated against COVID-19, although less than half of the sample was favorable to vaccinations and agreed with mandatory immunization. Acceptance of COVID-19 vaccination was found to be positively related with higher educational level (OR = 1.875, CI95% = 1.113–3.161; *p* = 0.018) and having social/mass media as a main source of information (OR = 2.415 CI95% = 1.358–4.296, *p* = 0.003). On the contrary, an inverse relationship was found between acceptance of COVID-19 vaccination and having fulfilled the questionnaire after the introduction of *green pass* (OR = 0.218, CI95% = 0.129–0.369; *p* < 0.001). Therefore, although this evidence needs to be further confirmed, it is possible to agree with previous studies reporting that compulsory measures, such as *green pass* implementation, must be accompanied by effective education and information strategies of the target population.

## 1. Introduction

Since the end of 2019, the spread of the severe acute respiratory disease caused by the novel coronavirus (SARS-CoV-2) has had great repercussions not only on people’s health and quality of life, but also on healthcare and socioeconomic systems [1]. While this paper is written, the Coronavirus Disease 2019 (COVID-19) has caused approximately 230 million cases and about 4.7 million deaths worldwide [2].

In Italy, more than 4 million confirmed cases were registered to date and about 130,000 people died, especially among the elderly [2,3].

Based on scientific evidence in the control of infectious diseases [4,5,6], several measures and surveillance systems were adopted and enforced to limit the spread of the virus and its burden [7]. Restriction of movements, social distancing, and use of facial masks were among the main mandatory measures established by governments all over the world [7]. In the meantime, a large number of scientific and medical institutions were engaged to rapidly develop and test an effective vaccine, that was firstly released at the end of 2020 [8,9].

In Italy, a strategic immunization plan was timely introduced by the Ministry of Health, starting the first administration at the end of December 2020 [10,11,12]. In the first phase of the vaccination campaign, also due to a limited availability of doses, immunization was offered to health care and police personnel, together with elderly and fragile people [10,11]. Subsequently, the vaccination was offered to other working categories such as teachers and to lower age classes [12]. The immunization campaign required a notable economic and organizational effort by Italian institutions and authorities [12]. The vaccination campaign reached the rate of more than 4 million doses administered per week [13], and simultaneously, the incidence of COVID-19 decreased [14], leading the Italian government to the revocation of some control measures such as the suspension of the use of facial masks outdoors. A certification (called “*green pass*”) was released to vaccinated people, or individuals who were affected by the disease and were healed or, finally, those with a negative rapid antigen test performed within the past 48 h or a negative molecular test executed within the past 72 h [15]. Since July 1, this certification was adopted as digital COVID-19 immunization certificate for travels in Europe.

As expected for all immunization strategies, besides the increasing of vaccinated people, an increase in vaccine hesitancy, which consists in a delay in acceptance or a refusal of vaccination despite its availability [16], was observed [13]. Moreover, on March 2021, the administration of the Vaxzevria vaccine was precautionary suspended by the Italian Drug Agency (Agenzia Italiana del Farmaco (AIFA)) following some cases of thrombosis registered worldwide and also in Italy, leading to an increase in the awareness of possible vaccination adverse effects [17,18]. Moreover, in order to face the epidemiological emergency and to guarantee a safe practice of the social and economic activities, at the end of July, the Italian government also decided to make the *green pass* certificate mandatory since August 6 to access to different public places such as theatres, cinemas, closed restaurants and, overall, for travelling [19]. The *green pass* can be acquired in digital or paper versions, when people have been vaccinated, or recently tested negative or recovered from COVID-19. After the approval of the mandatory *green pass*, there has been a different reaction in Italy, ranging from strong protests, to a sudden increase of booking for vaccine appointments.

In this scenario, vaccine acceptance may play a fundamental role, and it must be monitored. In fact, it is demonstrated that control measures, such as domestic vaccine passports, may have detrimental effects on people’s autonomy, motivation, and willingness to get vaccinated [20]; therefore, it is necessary supporting individuals’ autonomous motivation through specific health promotion intervention and informative campaign. Anyway, such actions firstly require a precise assessment of the level of vaccine acceptance and its determinants, to better target the communication campaign in order to fight vaccine hesitancy [21,22]. Rapid surveys are important tools for tracking knowledge, behaviors and perceptions of the population during an epidemic [23,24]. With respect to Italy, previous studies have investigated the levels of COVID-19 vaccine acceptance among various segments of the population, such as health care workers, students, general population [18,21,23,25]. No targeted studies have been performed with regard to elderly, which represent the population group mainly affected by the disease and, at the same time, one of the main targets of the third “booster” dose.

In this scientific context, the present study is aimed to assess the acceptance of COVID-19 vaccination in a specific sample of older adults from Southern Italy during the national vaccination campaign.

## 2. Materials and Methods

### 2.1. Setting and Participants

With a land surface area of 19,347 km^2^ and around 4 million inhabitants, the Apulia region covers the greater part of south-eastern Italy. In this geographical context, a cross-sectional study was performed from June to August 2021 among elderly people aged ≥65 years old. Subjects were enrolled, by a convenience sampling, among those joining religious, recreational or cultural associations or living in facilities hosting self-sufficient elderly. People were invited to voluntarily participate in the survey by responding to an online questionnaire. The link to the web-based questionnaire was sent, via social media or by email, directly to elderly or through a reference person working in the facilities. In both cases, the maximum diffusion of the online questionnaire was asked.

With a target regional population of 891,42 over-65 years old [26], a sample of at least 384 people should have been enrolled to explore the selected variables, within 95% confidence level and a 5% margin of error, as previously reported [18].

The study was anonymously conducted following the previsions of the World Medical Association Declaration of Helsinki. The Scientific and Ethical Institutional Board of the Italian Inter University Research Centre “Population, environment and health” (CIRPAS) approved the protocol (approval number 0530_2021, released on 23 June 2021).

### 2.2. Questionnaire

An online questionnaire, in the Italian language, was designed including two sections.

The first section was aimed to collect socio-demographic information such as gender, age, and educational level (none/elementary/middle/high/university degree). These items were designed based on a previous study in the same population [27] and the opinions of a panel of experts composed by one demographer, one epidemiologist, and one psychologist.

The second part included questions about their acceptance of both vaccinations in general and COVID-19 vaccines in particular. This part was structured by adapting the questionnaires used in previous studies to the specific age group [18,28]. The modified items included in this section were stated by a panel of experts including one public health expert, one epidemiologist, one psychologist and one biologist expert in infectious diseases prevention.

Participants were asked to:

1. Declare if they were favorable to vaccinations in general (yes/no);
2. Report if they had been immunized against the influenza virus in the season 2019/20 (yes/no);
3. Report if they had been immunized against the influenza virus in the season 2020/21 (yes/no);
4. Refer if COVID-19 vaccines may cause health problems (yes/no);
5. Report if they had received at least a single dose of a COVID-19 vaccine (yes/no);
6. Refer, if not yet vaccinated, if they were willing to be vaccinated against COVID-19 (yes/no);
7. Refer, if not yet vaccinated, if they were willing to be vaccinated against COVID-19 with any formulation (yes/no);
8. Refer, if not willing to be vaccinated, the main motivation (I don’t trust vaccines/these vaccines are not effective/I’m allergic/I’ve had the disease/I’m not at risk/the available vaccines may cause severe health consequences);
9. Refer if, in their opinion, the COVID-19 vaccination should be mandatory (yes/no);
10. Refer if they were favorable to the adoption of the “green pass” (yes/no);
11. Report their main source of information about COVID-19 vaccination (health care personnel, scientists/mass media/social media).

A previous pilot-study was performed on 54 elderly individuals in order to test the questionnaire’s validity (data neither published nor included in this paper). In order to evaluate the intelligibility of the questions, the subjects were asked to assign a score to each item of the questionnaire on a 7-point-scale (from 1: not meaningful to 7: very meaningful). Moreover, as reported in previous studies [28,29], in order to guarantee answers variability, the original questionnaire was modified in the pilot version: 10 further items (FI) reporting errors (grammatical and/or semantic) were added to the items (OI) belonging to the original questionnaire. OI reported a mean score for each question >6 (almost the maximum); FQ showed a mean score ≤1. These data confirmed that the content of the questionnaire was clear to the readers [28,29]. The reliability index was assessed for both the pilot and original study by using Cronbach’s alpha (internal consistency coefficient) [30,31]. The alpha values was 0.85 and 0.81, respectively, showing a satisfactory level of reliability [32].

Participants were recruited by approaching religious, recreational, residential and cultural facilities in each of the 6 provinces of the Apulia region and asking to involve attendances and other relatives and acquaintances. The percentage of enrolled people for provinces respected the stratification for provinces of the regional over 65 years population according to the last census [33]. In order to not exclude older people with limited access to the internet, a paper questionnaire was also distributed.

### 2.3. Statistical Analyses

A descriptive analysis was performed on respondents’ sociodemographic characteristics and on answers regarding experience, acceptance, and opinions about COVID-19 vaccination. Age was expressed as mean value ± standard deviation (SD). The other characteristics and answers were reported as number and percentage of respondents. The chi-squared test was carried out to highlight possible relationships between socio-demographic features and attitude of participants toward COVID-19 vaccination. Differences between answers given before and after the mandatory adoption of *green pass* (August 6) were also evaluated through chi squared test, without attributing any effects of the compulsory introduction of *green pass* to the possible changes in questions’ answers.

A logistic regression analysis was performed in order to identify possible factors associated to COVID-19 vaccine acceptance. In particular, educational level (elementary/middle school = 0; high school/degree = 1), source of information (health care personnel, scientists = 0; mass media/social media = 1), and date of fulfilling the questionnaire (before the mandatory adoption of green pass = 0; after the mandatory adoption of green pass = 1) were considered as independent variables, controlling for gender and age. Vaccine acceptance was considered as the dependent variable (expressed as 0 = respondents who had not yet been vaccinated against COVID-19 and were not willing to be immunized and 1 = people who had been vaccinated or were favorable to vaccination). Results were expressed as Odds Ratios (ORs) with corresponding 95% Confidence Intervals (95% CI).

A *p* value of 0.05 was assumed as significance level.

The software IBM SPSS version 27 for Windows (IBM Corp., Armonk, NY, USA) was used for the analysis.

## 3. Results

A total of 1041 completed questionnaires were fulfilled and collected, of which 59.7% and 40.3% before and after the *green pass* implementation, respectively. All questionnaires were completed online and no paper questionnaires were returned. The sample was mainly composed by females (58.3%) and high school educated (49.5%) subjects. The mean age of participants was 76.6 ± 6.5.

Table 1 shows the answers provided by participants about their attitude towards COVID-19 vaccination.

Overall, 901 (86.6%) participants were yet vaccinated against COVID-19; moreover, 64 of those who were not vaccinated were willing to be vaccinated in the future. Therefore, the total proportion of participants vaccinated or willing to be vaccinated was 92.7% (Table 1). Among those people who were neither vaccinated nor willing to be vaccinated, 15 (19.7%) reported as their main motivation for vaccine refusal to have already had the disease.

Table 2 shows the main sociodemographic characteristics of the sample and the differences between those who accepted COVID-19 vaccination and those who did not, with corresponding *p* values.

Table 3 reports the answers regarding COVID-19 vaccines provided by respondents grouped on the basis of their declared vaccine acceptance.

Less than half of the sample declared the acceptance of vaccines in general. However, the percentage of people reporting to have been vaccinated against influenza increased from 25.8% to 73.8% in the 2019/20 and 2020/2021 season, respectively. Moreover, the 87% thought that COVID-19 vaccines may cause health problems, but the 82% of the sample thought that the available formulations may have different side effects. Nevertheless, around 87% of the sample is already vaccinated against COVID-19. With regard to non-yet-vaccinated participants, a 45.7% declare the willingness to be vaccinated, but the remaining 54.3% (7.3% of the whole sample) declared they do not want to get vaccinated at all. Furthermore, none of the non-vaccinated people were available to be vaccinated with any of the COVID-19 vaccines. The main reported reason for immunization refusal was the opinion about the effectiveness of the available vaccines, followed by having previously had the COVID-19 and the fear of the severe health effects following the immunization. The 45.5% of participants thought that COVID-19 vaccination should become mandatory, but a lower percentage (33.3%) was favorable to the adoption of the *green pass*. Healthcare personnel and scientists were reported as the main sources of information about COVID-19 vaccination.

As for the comparison between willing and non-willing to be vaccinated, significant greater proportions of females, people older than 77 years, highly educated, and vaccinated against influenza in the previous and in the current season were found among vaccinated/willing to be vaccinated against COVID-19 people. As for their opinions, acceptance of COVID-19 vaccination was found to correlate with being favorable to vaccinations in general, with not recognizing possible health consequences of vaccines, nor differences in the side effects among the different vaccines formulations. Although the majority of vaccinated/willing to be vaccinated against COVID-19 individuals agreed with a possible mandatory immunization campaign, these results were not found to be statistically significant. As for the source of information, mass media account for the higher proportion of respondents who declared to accept COVID-19 vaccines.

With regard to the comparison between the period before and after the mandatory adoption of *green pass*, the results statistically significant are the following:

- The percentage of individuals vaccinated/willing to be vaccinated changed from 96% to 87.9% (*p* value < 0.001);
- The proportion of respondents who were favorable to vaccinations in general changed from 51.9% to 35.2% (*p* value < 0.001);
- The percentage of those who thought that COVID-19 vaccines may cause health problems changed from 80.7% to 96.4% (*p* value < 0.001);
- The percentage of participants who thought that COVID-19 vaccination should be compulsory changed from 64.9% to 16.9% (*p* value < 0.001);
- The percentage of those who were favorable to the *green pass* changed from 44.9 to 16.2% (*p* value < 0.001).

**Figure 1:**
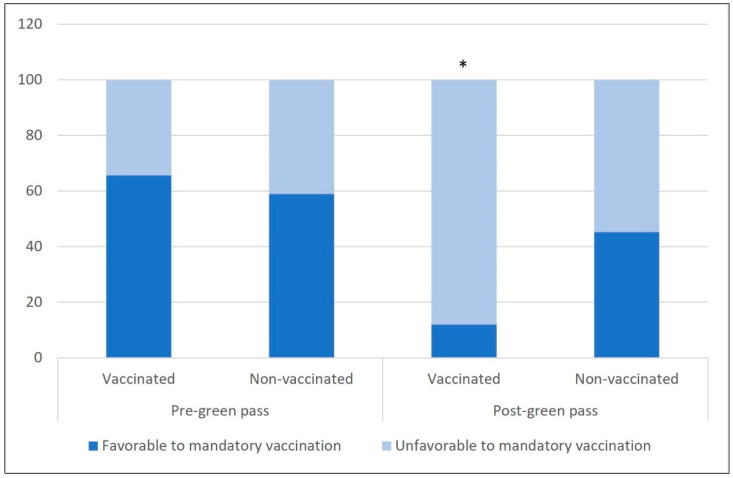
Proportions of respondents favorable or not to mandatory COVID-19 vaccination among vaccinated/non vaccinated respondents enrolled before and after the adoption of *green pass*. * *p* value < 0.05.

Figure 1 shows the amounts of vaccinated/non-vaccinated respondents who were or were not favorable to mandatory vaccination, before and after the introduction of the *green pass*.

It is possible to observe a significantly higher percentage of people non-favorable to mandatory vaccination among vaccinated individuals who took part in the study after the adoption of the *green pass* measure.

The results of the logistic regression analysis are shown in Table 4.

The analysis confirmed the relationship between vaccine acceptance and educational level, source of information and date of fulfilling the questionnaire. Specifically, having a higher educational level, reporting mass/social media as a main source of information and having completed the questionnaire before the introduction of the *green pass* as a compulsory measure were associated with vaccine acceptance. With regard to this, it should be noted that only three persons indicated “social media” as the main source of information.

## 4. Discussion

This study was carried out to assess the acceptance of COVID-19 vaccination in a sample of older adults in southern Italy, in the late course of the immunization campaign performed in Italy. The findings of the survey show a high proportion of individuals vaccinated or willing to be vaccinated against SARS-CoV-2 (92.7%). However, some contradictions emerged during the analysis of the results. First, the percentage of individuals who declared a general vaccine acceptance was low (45.1%) and notably lower than that of people who were immunized against COVID-19 (86.6%). It seems, therefore, that a great part of the sample may have undergone COVID-19 vaccination reluctantly. This seems consistent with previous findings: Biasio et al. reported that among those who stated a willingness to be vaccinated, there are hesitations and doubts, which are likely to increase because of the quantity and complexity of information available about the efficacy and safety of vaccines coming into use, which is often very technical and contradictory [23]. An example is given by the information delivered with regard to some cases of thrombosis registered worldwide and possibly related to a COVID-19 commercialized vaccine, that lead also the Italian Drug Agency (AIFA) to a precautionary and temporary suspension of the Vaxzevria COVID-19 vaccine [17]. This event significantly increased the perception of negative vaccine health consequences [18].

The proportion of respondents vaccinated or willing to receive COVID-19 vaccination in our study is higher than that registered in young adults in another Italian region (Emilia Romagna) by Reno et al. (68.9%) [34] and at national level by Barello et al. (86.1%) [35]. Additionally, Del Riccio et al. reported a lower level (81.9%) of intention to be vaccinated in a sample of Italian adults [21]. On the contrary, our findings are in line with the vaccine acceptance found, more recently, in a national study enrolling Italian adults (92%), and in another study on young adults from southern Italy (91.9%) [18].

With regard to age, a large international study demonstrated the role of age in vaccine acceptance: higher acceptance was reported in people aged ≥50 than in younger respondents [36]. Our study was the first conducted among elderly people (≥65 years) in Italy, although this population is among the first categories experiencing the third dose of the COVID-19 vaccine. Higher age was positively related with vaccine acceptance. A study conducted in southern Switzerland among over 65-years-old demonstrated a willingness to be vaccinated against COVID-19 in more than half of the sample, less than in our sample [37]. In this study, the main concerns reported by enrolled elderly were about both safety and efficacy [37]. It should be noted that in our study, despite the public debate regarding the possible side effects of COVID-19 vaccines, the main reason for vaccine hesitancy given by our sample was the lack of confidence in the efficacy of the available vaccines. Another study performed in United States reported a 91.3% of willingness to be vaccinated in a large sample of over 65-years-old people; a lower willingness was registered in women and black respondents, that reported the need to talk with their health providers before a decision [38]. This highlights, once again, the role of health care personnel in the delivery of correct information to their patients, addressing vaccine hesitancy. Moreover, the main source of information reported in our study were healthcare personnel and scientists. Therefore, health care policy may use healthcare providers as a key leverage point and ensure appropriate resources for them to reach and educate this older population at risk for vaccine hesitancy.

Nevertheless, we found that vaccine acceptance was related with high educational level and mass/social media as a main source of information. This also suggests that the promoting messages diffused through media were acknowledged by this elderly population, especially among those who had a higher education.

We also found significantly higher rates of reported vaccination and vaccine willingness in females compared to males as previously reported by Biasio et al. [23], but not consistent with other studies [21,38]. Socioeconomic factors, including higher education, were also associated with increased willingness to be vaccinated, similarly to other studies [38,39,40].

With regard to influenza vaccination, an increase of about 50% in vaccinated people was registered in the season 2020/21 with respect to the previous one, supporting the hypothesis that the efforts made by the Italian healthcare system in this direction were successful [18,41].

About half and one third of the sample thought that COVID-19 vaccines should be administered and are favorable to the adoption of the *green pass*, respectively. Notably, both these last proportions, such as those related to vaccine trust and acceptance, were significantly lower in the subject enrolled after the mandatory implementation of the *green pass*. Moreover, a higher proportion of participants who were unfavorable to mandatory vaccination was found among vaccinated ones who completed the questionnaire after the mandatory introduction of the *green pass*. It should be noted that the comparison of answers before and after the mandatory implementation of the *green pass* was performed on two different groups of participants; therefore, caution must be exercised in attributing any effect of this mandatory measure on the difference in question answering. Nevertheless, the logistic regression analysis also confirmed a relationship between vaccine acceptance and date of questionnaire fulfilling. Moreover, a previous study demonstrated that domestic vaccine passports may have detrimental effects on people’s motivation and willingness to get vaccinated [20]. In a recent study, performed in Germany by Graeber et al., 51% of the sample was in favor of a policy of mandatory vaccination against SARS-CoV-2 [42], and the approval rate for mandatory vaccination was significantly higher among those who would get vaccinated voluntarily (around 60%) than among those who would not get vaccinated voluntarily (27%). In Italy, as in other countries, the decision whether or not to get vaccinated against COVID-19 in the course of the pandemic has been left to the citizens. However, vaccination surveillance showed so far that the willingness to get vaccinated might not be sufficiently high to achieve a herd immunity. In this scenario, the Italian government chose to adopt the mandatory *green pass* to ensure the safe recovery of social and economic activities from COVID-19 pandemic. However, it is demonstrated that mandatory measures such as the compulsory adoption of a vaccine passport may be interpreted as a threat to human rights and civil liberty, and then decrease vaccine acceptance [43]. Therefore, interventions supporting individuals’ autonomous motivation to get vaccinated should be programmed and implemented beside compulsory previsions of law, even in non-pandemic times [20,43]. The early development of national public health laws and policies to provide a proportionate and graduated approach to compulsory vaccination in the context of a global health crisis, beside effective information campaigns, may represent an effective preparedness strategy [20,43].

This study has some limitations. First, participants were enrolled by a convenience sampling in a region from southern Italy; therefore, the final sample is not representative of the whole population of the Italian elderly. This aspect should be also considered in light of both socio-economic and COVID-19 epidemic features differences among the different areas of Italy. Moreover, vaccine acceptance has not been investigated in depth, in order to avoid an excessive length of the questionnaire. In particular, the knowledge about the available vaccines and the immunization mechanisms were not explored, such as other important factors influencing vaccine acceptance or refusal (e.g., having already had the diseases, changes in the epidemiological situation, etc.). This could have hidden important information; therefore, future researches should better address this issues along the evolution of the pandemic and vaccination campaigns.

However, this is the first study that offers a picture of vaccine acceptance and related factors in a large sample of Italian elderly during the first immunization against COVID-19. Although this evidence needs to be further confirmed, it allows us to highlight some aspect useful to improve the strategies of vaccination promotion in this specific age group.

## 5. Conclusions

Vaccination is the most efficient control measure against the current COVID-19 pandemic. Our study demonstrated a high level of over 65-year-old people vaccinated/willing to be vaccinated against COVID-19; however, a lower vaccine acceptance was associated to the date of questionnaire fulfilling (after the mandatory implementation of the *green pass*). Therefore, although this evidence needs to be further confirmed, it is possible to agree with previous studies reporting that compulsory measures must be accompanied by effective education and information strategies of the target population [20,43], paying attention to the spread of data not supported by scientific evidence [18]. In this context, the role of reference healthcare personnel is crucial. At the same time, communication campaigns based on mass media should be tailored to those categories who were shown to be more hesitant.

Data obtained from the present study may be useful to policy makers to improve the COVID-19 control strategies, especially in light of the third “booster” dose targeted primarily to vulnerable groups and older people.

## Figures and Tables

**Table 1:** Answers related to COVID-19 vaccination provided by participants.

Item	*n* (%)
Vaccinated against COVID-19	
Yes	901 (86.6)
No	140 (13.4)
If not vaccinated, willing to be vaccinated against COVID-19	
Yes	64 (45.7)
No	76 (54.3)
If not vaccinated, willing to be vaccinated against COVID-19 with any	
formulation	
Yes	0 (0)
No	140 (100)
If neither vaccinated nor willing to be, main motivation for refusal:	
I don’t trust vaccines	3 (3.9)
The available vaccines are not effective	48 (63.2)
I’m allergic	0 (0)
I’ve had the disease	15 (19.7)
I’m not at risk	1 (1.3)
The available vaccines may cause severe health consequences	9 (11.8)

**Table 2:** Socio-demographic characteristics of participants on the whole and grouped by willingness/unwillingness to be vaccinated.

Item	Participants *n* = 1041	Vaccinated/Willing to Be Vaccinated *n* (%)		*p* Value
		Yes	No	
Gender				0.024
males	434 (41.7)	393 (90.6)	41 (9.4)	
females	607 (58.3)	572 (94.2)	35 (5.8)	
Age				<0.001
≤76 years	553 (53.1)	487 (88.1)	66 (11.9)	
≥77 years	488 (46.9)	47 8 (98)	10 (2)	
Educational level				<0.001
Elementary	28 (2.7)	26 (92.9)	2 (7.1)	
Middle	310 (29.8)	277 (89.4)	33 (10.6)	
High school	515 (49.5)	496 (96.3)	19 (3.7)	
Degree	188 (18.1)	166 (88.3)	22 (11.7)	

**Table 3:** Answers regarding vaccinations and COVID-19 vaccination provided by respondents on the whole and grouped by willingness/unwillingness to be vaccinated.

Item	Respondents *n* (%)	Vaccinated/Willing to Be Vaccinated *n* (%)		*p* Value
		Yes	No	
Favorable to vaccination in general				<0.001
Yes	470 (45.1)	460 (97.9)	10 (2.1)	
No	571 (54.9)	505 (88.4)	66 (11.6)	
Vaccinated against influenza (season 2019/20)				0.004
Yes	269 (25.8)	260 (96.7)	9 (3.3)	
No	772 (74.2)	705 (91.3)	67 (8.7)	
Vaccinated against influenza (season 2020/21)				<0.001
Yes	768 (73.8)	726 (94.5)	42 (5.5)	
No	273 (26.2)	239 (87.5)	34 (12.5)	
Might the COVID-19 vaccines cause health problems?				0.015
Yes	906 (87)	833 (91.9)	73 (8.1)	
No	135 (13)	132 (97.8)	3 (2.2)	
Might all the COVID-19 vaccines have the same side effects?				0.001
Yes	188 (18.1)	185 (98.4)	3 (1.6)	
No	853 (81.9)	780 (91.4)	73 (8.6)	
Should COVID-19 vaccination become mandatory?				0.388
Yes	474 (45.5)	443 (93.5)	31 (6.5)	
No	567 (54.5)	522 (92.1)	45 (7.9)	
Favorable to the adoption of the “*green pass*”				0.4
Yes	347 (33.3)	325 (93.7)	22 (6.3)	
No	694 (66.7)	640 (92.2)	54 (7.8)	
Main sources of information about COVID-19				0.009
vaccination				
Healthcare personnel, scientists	636 (61.1)	577 (90.7)	59 (9.3)	
Mass media (i.e., television, magazines)	402 (38.6)	385 (95.8)	17 (4.2)	
Social media (i.e., Facebook, Twitter, Instagram, WhatsApp)	3 (0.3)	3 (100)	0 (0)	

**Table 4:** Results of the logistic regression model built considering the acceptance of COVID-19 vaccination as the outcome.

Independent Variable	Vaccine Acceptance
	OR (CI 95%)
Educational level	
Elementary/middle	Reference
High school/degree	1.875 (1.113–3.161) *
Source of information	
Health care personnel, scientists	Reference
Mass media/social media	2.415 (1.358–4.296) **
Date of fulfilling the questionnaire	
Before the mandatory adoption of *green pass*	Reference
After the mandatory adoption of *green pass*	0.218 (0.129–0.369) **

OR (CI 95%): odds ratio (95% confidence interval); * *p* < 0.05; ** *p* < 0.01.

## Data Availability

All data presented are available upon request from the corresponding author (F.G.).
